# An Unrecognized High Incidence of Asymptomatic Uterine Torsion in Pregnancies with Adenomyosis that Complicate Cesarean Delivery

**DOI:** 10.1007/s43032-025-02045-9

**Published:** 2026-01-08

**Authors:** Yuri Yoshida, Takayuki Iriyama, Yu Ariyoshi, Haruka Matsui, Kensuke Suzuki, Ayako Hashimoto, Mari Ichinose, Masatake Toshimitsu, Seisuke Sayama, Takahiro Seyama, Kenbun Sone, Keiichi Kumasawa, Osamu Wada-Hiraike, Yutaka Osuga, Yasushi Hirota

**Affiliations:** Department of Obstetrics and Gynecology, Faculty of Medicine, University of Tokyo, 7-3-1 Hongo, Bunkyo-ku, Tokyo, 113-8655 Japan

**Keywords:** Adenomyosis, Cesarean delivery, Focal type, Uterine torsion

## Abstract

The association between adenomyosis and uterine torsion, a rare, serious obstetric condition, remains unclear and has not been systematically investigated. This study aimed to elucidate the incidence, risk factors, and clinical characteristics of uterine torsion in pregnancies complicated by adenomyosis. We conducted a retrospective cohort study of women with adenomyosis undergoing cesarean section at The University of Tokyo Hospital from 2010 to 2023. Adenomyosis was diagnosed via magnetic resonance imaging and/or ultrasonography before and/or during early pregnancy. Uterine torsion was defined as > 45° rotation around the long uterine axis during cesarean section. We compared the clinical characteristics, adenomyotic features and surgical details of women with and without uterine torsion. Among 135 pregnant women with adenomyosis, 70 underwent cesarean section. Within this cesarean delivery cohort, uterine torsion was identified in six (8.6%) women. All torsion cases were asymptomatic and involved focal type adenomyosis; these six cases represented 13.6% (6/44) of the 44 women with focal type adenomyosis in this cesarean cohort. The torsion group required supraumbilical skin incisions (50.0% [3/6] vs. 0% [0/64], *P* < 0.001) and atypical uterine incisions (83.3% [5/6] vs. 23.4% [15/64], *P* = 0.006) significantly more often. This study highlights a high incidence (8.6%) of asymptomatic uterine torsion in a cohort of women with adenomyosis undergoing cesarean delivery. These findings suggest that focal adenomyosis may be a key risk factor and generate an important hypothesis that requires further investigation. Surgical planning should consider the possibility of uterine torsion, which may require tailored intraoperative strategies involving non-standard incisions.

## Introduction

Uterine torsion, defined as a rotation of the uterus by more than 45° around its long axis, is a rare event during pregnancy [1, 2]. Typical symptoms include acute abdominal pain and vaginal bleeding [2–4], although approximately 10% of cases are asymptomatic and may be discovered incidentally during cesarean section [3]. Known risk factors for uterine torsion include uterine fibroids, pelvic tumors, and adhesions from previous surgeries [3]. In particular, uterine torsion in pregnancies complicated by fibroids has been frequently reported [2, 5–8], with some studies indicating that 20%–30% of uterine torsion cases involve coexisting fibroids [9].

Adenomyosis, a condition characterized by the presence of ectopic endometrial tissue within the myometrium, has recently gained attention for its association with adverse perinatal outcomes such as miscarriage, preterm birth, and preeclampsia [10–17]. Similar to uterine fibroids, adenomyosis can alter uterine morphology and size, potentially affecting the biomechanical properties of the pregnant uterus. However, knowledge regarding uterine torsion in pregnancies complicated by adenomyosis is exceptionally limited, with findings largely confined to a single case report by Kitada et al. [18]. Consequently, the true clinical picture of uterine torsion in this population remains unclear.

In this study, we aimed to determine the incidence of uterine torsion in pregnancies complicated by adenomyosis, investigate the association between the characteristics of adenomyotic lesions (e.g., extent, location, and size) and the risk of uterine torsion, and evaluate the impact of uterine torsion on perinatal management, including cesarean delivery techniques.

## Materials and Methods

This retrospective cohort study was conducted at the University of Tokyo Hospital, a tertiary care university hospital in Japan, with the approval of the Institutional Review Board (Approval No. 3053-1). The study used an opt-out method to obtain patient consent, with information disclosed on the hospital website, following the guidelines of the ethics committee.

We included pregnant women with adenomyosis who underwent perinatal management and delivered at or after 12 weeks of gestation at our institution between January 2010 and December 2023. Patients with multiple pregnancies, fetal abnormalities, or uterine malformations were excluded.

Adenomyosis was diagnosed on the basis of magnetic resonance imaging (MRI) and/or transvaginal ultrasonography findings obtained before pregnancy and/or early pregnancy. MRI diagnosis was based on either (1) the presence of a myometrial mass with indistinct margins and primarily low signal intensity or (2) diffuse or focal thickening of the junctional zone, forming an ill-defined area of low signal intensity on T2-weighted images [19]. Ultrasound diagnosis adhered to the morphological uterine sonographic assessment (MUSA) criteria established in 2015 [20–22], including features such as asymmetrical thickening, myometrial cysts, hyperechoic islands, fan-shaped shadowing, echogenic subendometrial lines and buds, translesional vascularity, and an irregular or interrupted junctional zone. Before 2015, the diagnosis of adenomyosis was retrospectively confirmed in the medical records according to the MUSA criteria.

Adenomyotic lesions in patients meeting the diagnostic criteria were classified based on the extent and location of imaging findings [21, 23, 24]. Regarding the extent, lesions were categorized as focal type if they were predominantly localized with at least 25% of the lesion circumference surrounded by normal myometrium or as diffuse type if lesions were widespread with less than 25% surrounded by normal myometrium [21, 24]. The lesion location was classified as extrinsic type (lesions predominantly located on the serosal side of the uterus), intrinsic type (lesions abutting the endometrium), or indeterminate type (lesions extending throughout all myometrial layers or multiple lesions) [23, 24]. The maximum diameter of the adenomyotic lesion was measured on transvaginal ultrasound performed before or during early pregnancy. Transvaginal ultrasound examinations were conducted by obstetrician-gynecologists, and the resultant images were digitally archived as Digital Imaging and Communications in Medicine (DICOM) data within the institution’s image management system (Claio). Uterine ultrasound images were retrospectively analyzed by a specialist certified by the Japan Society of Ultrasonics in Medicine. The specialist, who was blinded to the perinatal outcomes, classified adenomyotic lesions based on their extent, location, and maximum diameter.

Uterine torsion was diagnosed based on intraoperative findings during cesarean section and was defined as a rotation of the uterus by 45° or more around its long axis. The degree of rotation (angle), direction of rotation, and presence or absence of associated symptoms were extracted from the patients’ medical records.

Clinical information was collected from patient medical records, including maternal baseline characteristics (maternal age, parity, body mass index [BMI], mode of conception, smoking history), perinatal outcomes (gestational week at delivery, mode of delivery, birth weight, 5-min Apgar score, umbilical artery pH, admission to neonatal intensive care unit [NICU]), characteristics of adenomyosis (extent, location, maximum diameter), and details of cesarean section (estimated blood loss, surgical time, type of skin incision, type and location of uterine incision).

Statistical analyses were performed using EZR (Easy R) version 1.62 [25]. Continuous variables are expressed as the median and interquartile range (IQR) and were compared between groups using the Mann–Whitney U test. Categorical variables are expressed as frequencies and percentages and were compared using Fisher’s exact test. Statistical significance was set at *P* < 0.05.

## Results

**Fig. 1:**
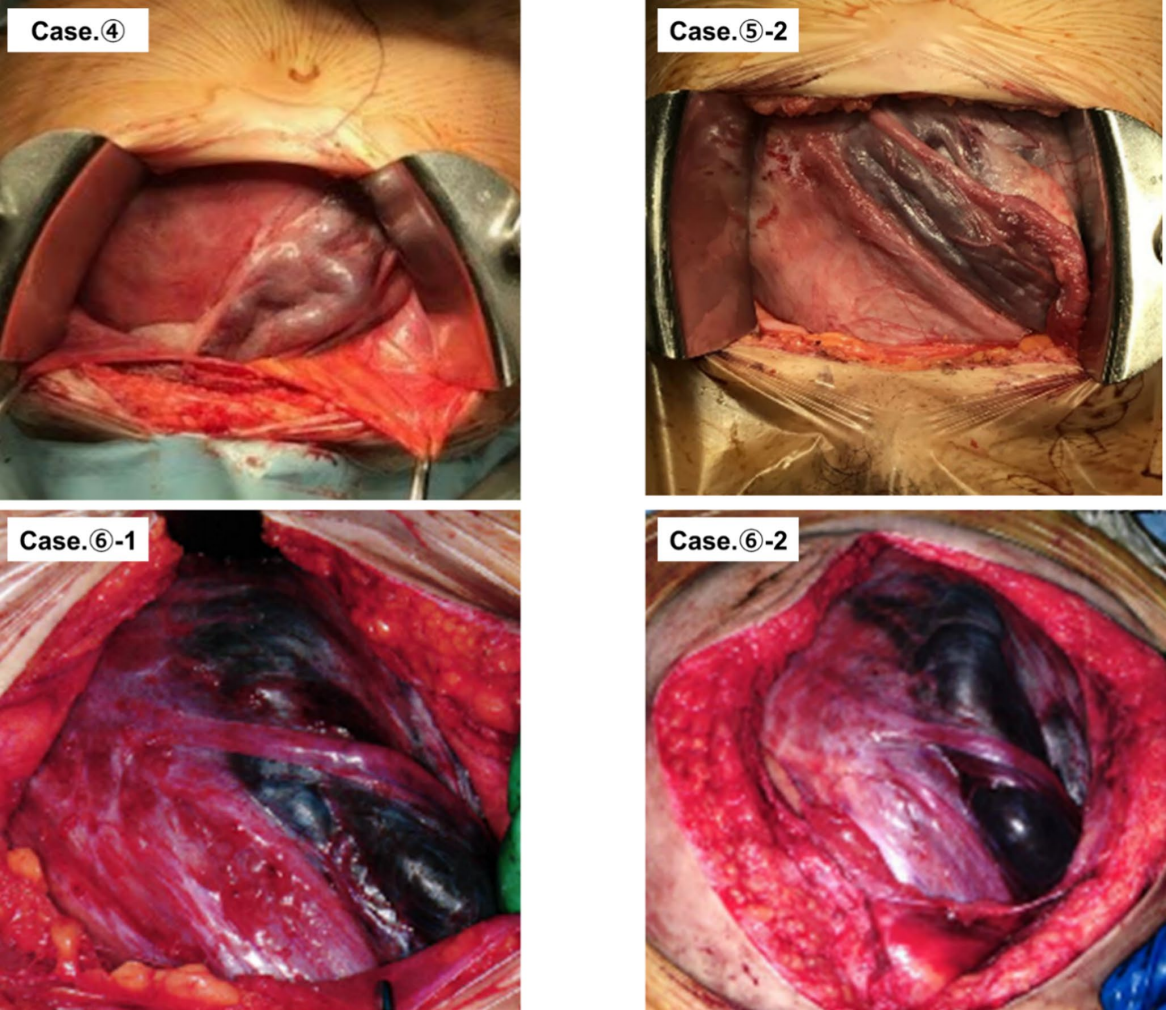
Representative intraoperative photographs of pregnancies complicated by uterine torsion. These photographs show the surgical field during cesarean delivery in patients with uterine torsion

Between January 2010 and December 2023, 135 pregnant women with adenomyosis delivered at our hospital after 12 weeks of gestation. Of these, 70 underwent cesarean section. Intraoperative uterine torsion was confirmed in six (8.6%) of these 70 women. Notably, two of these six patients experienced uterine torsion in two consecutive pregnancies. A clinical summary of the six patients (representing eight pregnancies with torsion) is presented in Table 1, and Fig. 1 shows representative intraoperative photographs of the cases with available records. All cases of uterine torsion were asymptomatic, with no preceding clinical symptoms such as abdominal pain or vaginal bleeding. In addition, none of the patients with torsion had coexisting uterine fibroids. Furthermore, none of the cases had suspicious findings of torsion on routine antenatal ultrasound examinations; torsion was diagnosed incidentally for the first time at the time of cesarean section. As shown in Fig. 1, in Cases 4, 5 − 2, 6 − 1, and 6 − 2, the uterus was rotated by 90°–100°. Due to this significant rotation, anatomical structures normally found on the posterior or lateral aspects of the uterus, such as the uterine venous plexus, round ligaments, and fallopian tubes, were observed in the anterior positions. Consequently, these structures appeared immediately beneath the surgical incision after opening the peritoneum. In Case 4, torsion caused the posterior uterine wall to rotate fully to the anterior position, leading to an inadvertent incision of the posterior wall when the anterior uterine incision was made. Although intraoperative photographic records were not available for Cases 1, 3, and 5 − 1, similar findings of significant uterine torsion and anatomical displacement were documented in these cases. Furthermore, all patients with uterine torsion experienced difficulty in exposing the incision site.

**Table 1:** Summary of maternal background characteristics in pregnancies complicated by uterine torsion

Case No.	Age	Parity	Delivery week	Indications for cesarean section	Rotation
①	35	1	36^+ 4^	Previous uterine surgery	Left 90°
②	40	0	34^+ 5^	Non-reassuring fetal status	Right 60°
③	37	0	38^+ 1^	Breech presentation	Left 75°
④	42	0	36^+ 4^	Placenta previa	Left 100°
⑤−1	34	0	36^+ 2^	Non-reassuring fetal status	Right 90°
⑤−2	36	1	37^+ 5^	Previous cesarean section	Right 90°
⑥−1	37	0	37^+ 5^	Transverse presentation	Right 90°
⑥−2	39	1	37^+ 3^	Placenta previa	Right 90°

Maternal baseline characteristics, perinatal outcomes, characteristics of adenomyotic lesions, and intraoperative details of cesarean section were compared between the group with uterine torsion (torsion group, *n* = 6) and the group without uterine torsion (non-torsion group, *n* = 64). The results are summarized in Table 2. There were no significant differences in maternal baseline characteristics or perinatal outcomes between the two groups.

**Table 2:** Comparison of maternal baseline characteristics, perinatal outcomes, adenomyotic lesion characteristics, and intraoperative details of caesarean section between pregnant women with adenomyosis in the torsion and non-torsion groups

Maternal and Neonatal Characteristics	Uterine torsion (*n*=6)	Non-torsion (*n*=64)	*P* value
Age (y), median (IQR)	37.0 (35.5-39.3)	38.0 (35.8-40.0)	0.74
Primiparity (%)	1 (16.7%)	11 (17.2%)	>0.99
BMI, median (IQR)	21.2 (21.0-24.6)	20.8 (19.2-23.5)	0.25
ART (%)	5 (83.3%)	18 (39.1%)	0.013
Delivery week, median (IQR)	36.0 (36.0-36.8)	37.0 (34.8-37.0)	0.81
Birth weight (g), median (IQR)	2499 (2112-2792)	2525 (2056-2897)	0.88
5min Apgar score, median (IQR)	9 (9-9)	9 (8-9)	0.26
UApH, median (IQR)	7.25 (7.22-7.28)	7.28 (7.25-7.31)	0.12
NICU admission	2 (33.3%)	21 (32.8%)	>0.99
Characteristics of adenomyosis lesions			
Extent of adenomyosis			
Focal	6 (100%)	38 (59.4%)	0.078
Diffuse	0 (0%)	26 (40.6%)	
Location of adenomyosis			
Intrinsic	1 (16.7%)	7 (10.9%)	0.57
Extrinsic	3 (50%)	28 (43.8%)	
Indeterminate	2 (33.3%)	29 (45.3%)	
Size of adenomyosis (cm), median (IQR)	7 (7-7)	5 (3-7)	0.26
Information about cesarean section			
Blood loss (ml), median (IQR)	1185 (828-1355)	1300 (989-2030)	0.25
Surgical time (min), median (IQR)	66.0 (52.3-92.5)	64.0 (52.0-82.0)	0.77
Skin incision			
Pfannenstiel (%)	0 (0%)	25 (39.1%)	<0.001
Midline vertical incision (%)	6 (100%)	39 (60.9%)	
Supraumbilical skin incision (%)	3 (50%)	0 (0%)	<0.001
Uterine incision			
Lower uterine transverse incision (%)	1 (16.7%)	49 (76.6%)	0.006
Others (%)	5 (83.3%)	15 (23.4%)	

*IQR* Interquartile range; *BMI* Body mass index; *ART* Assisted reproductive technology; *UApH* Umbilical artery pH; *NICU* Neonatal intensive care unit

Regarding the extent of adenomyotic lesions, all six women (100%) in the torsion group had focal adenomyosis. Of the 44 women with focal type adenomyosis in the entire cesarean section cohort, six (13.6%) developed uterine torsion. Regarding lesion location, in the torsion group, 16.7% (1/6) were intrinsic type, 50% (3/6) were extrinsic type, and 33.3% (2/6) were indeterminate type. In the non-torsion group, these proportions were 10.9% (7/64) intrinsic type, 43.8% (28/64) extrinsic type, and 45.3% (29/64) indeterminate type, none of which were statistically significant. The median maximum lesion diameter was 7 cm (range, 3–11 cm) in the torsion group and 5 cm (range, 2–12 cm) in the non-torsion group, with no significant differences observed.

**Fig. 2:**
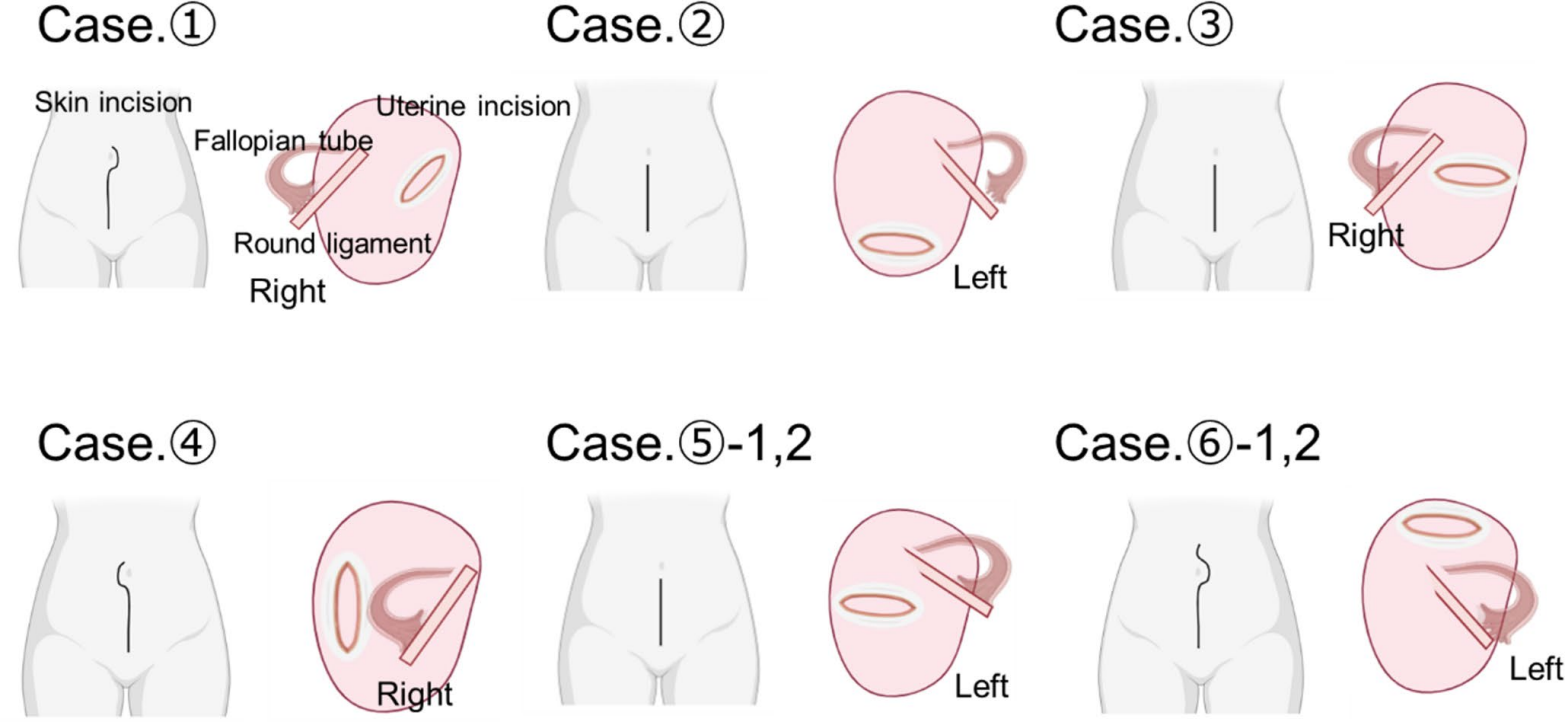
Schematics of skin and uterine incisions for pregnancies complicated by uterine torsion. The schematics are based on actual cesarean deliveries in eight pregnancies (six women) with uterine torsion. They depict varied surgical approaches and the corresponding abnormal orientation of the fallopian tubes and round ligaments (with laterality specified) due to uterine rotation. Uterine incision sites were selected after confirming areas free of adenomyotic lesions and the placenta using intraoperative ultrasonography. Created using BioRender https://www.biorender.com/

Regarding the details of cesarean section, there were no significant differences between the groups in estimated blood loss (median [interquartile range: IQR]: 1185 mL [828–1355 mL] vs. 1300 mL [989–2030 mL], *P* = 0.25) or surgical time (median [IQR]: 66 min [52.3–92.5 min] vs. 64 min [52.0–82.0 min], *P* = 0.77). However, regarding skin incisions, as shown in Fig. 2 and Table 2, a significantly higher proportion of patients in the torsion group required a midline vertical incision with supraumbilical extension than those in the non-torsion group (50% [3/6] vs. 0% [0/64], *P* < 0.001). Similarly, for uterine incisions, a significantly higher proportion of patients in the torsion group required atypical incisions that differed from the usual lower transverse approach than those in the non-torsion group (83.3% [5/6] vs. 23.4% [15/64], *P* = 0.006). As shown in Fig. 2, the following atypical uterine incisions were performed, as these were the only feasible approaches in these respective cases: a transverse/oblique incision of the uterine corpus in Case 1, corporeal transverse incisions in Cases 3 and 5, posterior wall vertical incision in Case 4, and transverse fundal incision in Case 6. Moreover, securing an adequate uterine incision site was extremely challenging for all patients in the torsion group. A retractor was placed, and lateral traction of the abdominal wall was applied by an assistant to adequately expose the appropriate surgical field for the uterine incision.

## Discussion

This is the first study to reveal a high incidence (8.6%) of asymptomatic uterine torsion within a specific cohort: pregnant women with adenomyosis who delivered via cesarean section. A key finding was that all cases of uterine torsion occurred in women with focal-type adenomyosis, suggesting that this subtype may be a significant, previously unrecognized risk factor. Furthermore, our results demonstrated that uterine torsion substantially complicates cesarean delivery, making standard approaches such as Pfannenstiel skin incisions and lower uterine segment transverse incisions exceptionally difficult and often requiring atypical, more extensive incisions.

These findings are particularly striking because uterine torsion during pregnancy is a rare condition, and its occurrence in pregnancies complicated by adenomyosis has, to date, been documented in only a single case report [18]. In stark contrast, our cohort study revealed that asymptomatic uterine torsion was identified in as high as 8.6% of women with adenomyosis undergoing cesarean section. This incidence is remarkably high, particularly considering that reports of uterine torsion during pregnancy are generally limited to sporadic cases [2, 4–8]. Moreover, previous literature suggests that uterine torsion during pregnancy typically presents acutely with severe symptoms, such as acute abdominal pain and vaginal bleeding, with asymptomatic cases considered relatively uncommon (approximately 10%) [3]. Therefore, our finding that all identified cases of uterine torsion in pregnancies complicated by adenomyosis were entirely asymptomatic is noteworthy.

These noteworthy findings have significant clinical implications. While adenomyosis is known to be associated with numerous obstetric complications [10–17, 24], its link with uterine torsion has remained unclear. Our study suggests that focal adenomyosis may be an independent risk factor for uterine torsion, offering new perspectives on understanding subtype-specific risks, whereas traditionally, diffuse adenomyosis has been considered high-risk [24]. This study also revealed that the presence of uterine torsion significantly complicates cesarean delivery procedures. In many cases, adequate surgical exposure for fetal delivery was difficult, necessitating an extension of the skin incision and requiring the use of retractors and lateral traction. Additionally, in five of six cases, a standard lower uterine segment transverse incision was not feasible. Considering these significant intraoperative complications, the potential for standard procedures to be compromised warrants careful preoperative consideration. For optimal surgical field exposure, a vertical skin incision is preferable, and patients should be informed about the potential need to extend the incision above the umbilicus. To circumvent challenges related to the uterine incision site, such as avoiding adenomyotic lesions or the placenta, intraoperative ultrasonography is crucial to determine the optimal incision site in real-time.

From a research perspective, the observation that all cases were asymptomatic suggests that the underlying mechanism of torsion in adenomyosis may differ from that in other conditions. In pregnancies with uterine fibroids or adnexal tumors, masses can destabilize the uterus, leading to acute, symptomatic rotation. In contrast, as the uterus enlarges during pregnancy, the normal myometrium may stretch disproportionately around the rigid, less distensible tissue of adenomyosis, potentially leading to a gradual, asymptomatic progression of torsion. This proposed mechanism is a hypothesis that could be explored in future biomechanical or prospective imaging studies. This gradual process could complicate preoperative image-based diagnosis; indeed, no cases in our study were reliably predicted using preoperative imaging. We utilized the established > 45° cutoff [1, 2] to define clinically significant torsion that is likely to impact surgical planning. Although milder, physiological rotations of less than 45° were present in our cohort, they were not included in this analysis as their pathological significance is considered low. In the future, serial imaging assessments at different time points may be useful for predicting the occurrence or progression of torsion, which remains an important area for investigation.

Although this study is the first to systematically investigate the surgical implications of uterine torsion in pregnancies with adenomyosis, it has several limitations. First, the number of uterine torsion cases (*n* = 6) was small, limiting the statistical power to identify risk factors. Second, a primary limitation is the study’s selection bias. As our diagnosis was based on intraoperative findings, our cohort was restricted to women who underwent cesarean delivery. Consequently, the observed 8.6% incidence cannot be generalized to the entire population of pregnant women with adenomyosis, as it excludes those who deliver vaginally. Furthermore, the difficulty in preoperative diagnosis made it challenging to identify torsion in advance. A prospective study to determine if there are any subtle imaging or clinical clues that could predict this complication preoperatively, even if it is asymptomatic, would be highly valuable. Overall, our current findings generate an important hypothesis that requires validation in larger, multicenter studies.

## Conclusion

In pregnancies complicated by adenomyosis, asymptomatic uterine torsion occurs at a high frequency of 8.6% among women undergoing cesarean section, with focal type adenomyosis emerging as a potentially significant risk factor. The presence of uterine torsion substantially complicates cesarean delivery, often rendering standard lower uterine segment approaches difficult and necessitating atypical surgical procedures. Therefore, careful preoperative evaluation and tailored intraoperative strategies, being mindful of the potential for uterine torsion, are crucial.
